# Mechanics of the Jump Shot: The “Dip” Increases the Accuracy of Elite Basketball Shooters

**DOI:** 10.3389/fpsyg.2021.658102

**Published:** 2021-06-28

**Authors:** Luke S. J. Penner

**Affiliations:** Department of Kinesiology, University of Manitoba, Winnipeg, MB, Canada

**Keywords:** basketball, jump shot, accuracy, NBA, NCAA

## Abstract

The present study assessed the mechanics of the basketball jump shot to determine whether or not the “dip” increased shot accuracy. There remained a debate between coaches who believed “dipping” was too slow and coaches who believed “dipping” increased accuracy. A mixed design was used for the present study with elite high-school and university players all performing shots with and without the “dip” at four distances: the last hash mark before the free throw line (3.125 m), the length of an imaginary hash mark beyond the free throw line (4.925 m), the top of the free throw circle (6.025 m), and the three-point line (6.750 m). These distances best emulated where the majority of shots were attempted in a game. Thirty-six athletes completed the study, with accuracy and shot quality being measured using Hardy-Parfitt’s six-point scale. The results of the present study indicated that the “dip” led to approximately a 7–9% increase in accuracy of the jump shot for both high school shooters, and university shooters, suggesting that coaches should begin to teach the “dip” in a player’s shooting motion to improve scoring results.

## Introduction

In basketball, the goal is to outscore the opponent by scoring more baskets. One of the most common techniques to score is the jump shot (Knudson, 1993; Gryko et al., 2018; Okazaki and Rodacki, 2018). In order to optimize accuracy, a player must be efficient and consistent with his shooting motion. Therefore, an elite athlete will attempt to reduce movement variability, the noise that impacts performance, in order to achieve maximal success with shooting the basketball (Button et al., 2003).

Basketball is a dynamic sport, and as a result, there is a lot of variability that can impact a player’s shooting motion. Knudson (1993) states that there are six key biomechanical components to optimize shooting success: staggered stance and a vertical jump; aligned shooting plane to the basket; high point of release; proper angle of release; coordination of upper and lower limbs; and backspin on the ball. Identifying which components might be more impactful to improving any of these teaching points will ultimately help a team win a game. One such movement is the “dip” in the shooting motion of the jump shot.

The “dip” is defined as the movement of lowering the ball below a player’s shooting pocket, the area of the body when all parts of the shooting arm are in a vertical plane out in front of the shoulder holding onto the ball. By lowering the ball, the shooting fingers would leave the vertical plane, the “dip.” The “dip” decreases the chance of an angular release, a lateral movement from the shot path plane (Okubo and Hubbard, 2015). Without using the “dip,” an awkward lift to the player’s set point, the point at which the ball moves toward the basket, would occur. As a result, movement variability is reduced.

As a coach, it is important to recognize and understand one’s personal biases in what makes an effective jump shooter. Drysdale (1972) explained that until scientific evidence is provided to coaches, they will use their own interpretations and create their own hypotheses as to what makes an athlete a successful jump shooter. Each coach will focus on different components of the jump shot, from the “dip” to visual cues. These teaching points are often ones that are familiar to the coach. These different styles of teaching result in different styles of shooting, which may impact accuracy. This variety is often because the player develops a comfort for their specific shooting style. However, tweaks to a player’s shooting motion often occur, guided by a coach, as the player grows and develops. These decisions are always made in order to increase accuracy. Therefore, as more scientific evidence for different mechanics of the jump shot, like the “dip,” are researched, the more coaches will embrace and utilize the motion in their teachings.

**FIGURE 1:**
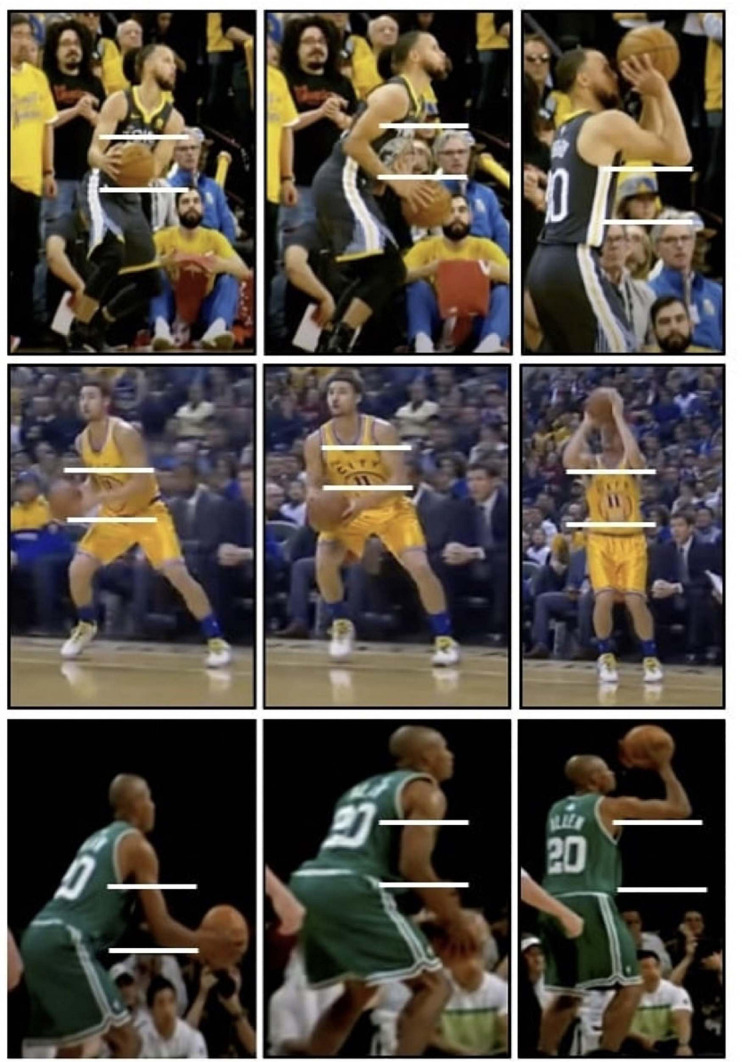
Demonstration of Elite Shooters Steph Curry, Klay Thompson, and Ray Allen “dipping” the basketball after catching the ball off a pass; the ball is caught in the “shot pocket” (indicated by white lines), lowered below it prior to raising the ball to the “set point,” and shooting the ball (the “dip”) (ShotMechanics, 2014; Splash Lab, 2016; Basketball Tips Shotur, 2019).

The game of basketball provides many variations of a situation, such as a catch and shoot, off a screen, off the dribble, and defenders. In order to isolate the “dip,” the catch and shoot scenario allows the most control for studying and will create a foundation for future studies with more variables. The natural form of a shooter, whether a “dipper” or “non-dipper,” will be easily identified. The “dip” is often seen as a poor shooting decision by coaches because of increased shooting time once the ball is caught by the player. There is a subset of coaches who believe in the value of “dipping” because of anecdotal evidence of increasing accuracy. This belief is often backed by the fact that many elite shooters, including Steph Curry, Klay Thompson, and Ray Allen are seen “dipping” the basketball prior to shooting (see Figure 1). However, no scientific data exists on the efficacy of “dipping” the basketball. Therefore, the aim of this study is to assess the relationship between “dipping” the basketball in one’s jump shooting motion and shooting accuracy, regardless of position and current shooting motion. The hypothesis is that “dipping” will result to higher shooting accuracy.

## Materials and Methods

### Participants

The study was promoted within the elite basketball community of Manitoba, using the Basketball Manitoba platform and the two major universities: University of Manitoba and University of Winnipeg. When a player expressed interest in the study, exclusionary criteria were used to obtain the most elite athletes. These criteria were:

1. The player had played at least 4 years in an elite setting which would include any combination of provincial, regional, club, or university experience.
2. The player had no pre-existing injury to the shoulder, wrist, hand, hip, knee, or ankle less than 6 months prior to the study date, which was self-reported.

As a result, there were 36 male subjects from elite basketball teams in Manitoba. They were assigned to one of two groups: university (*n* = 18); or high school (*n* = 18). The placement within the group was dependent on where the athlete last played basketball. All 18 university players played at the University of Manitoba, while 14 players played for the Manitoba Provincial team, and 4 played a combination of regional and club basketball. All athletes were self-declared as healthy without any pre-existing injury to the shoulder, wrist, hand, hip, knee, or ankle and had more than 4 years of elite basketball experience. Permission to conduct the present study was also granted by the Education/Nursing Research Ethics Board (ENREB) at the University of Manitoba. Individual players gave written informed consent prior to participation. While those under 18 years of age, had a parent or guardian provide written informed consent and the player provided written assent.

### Study Design

**FIGURE 2:**
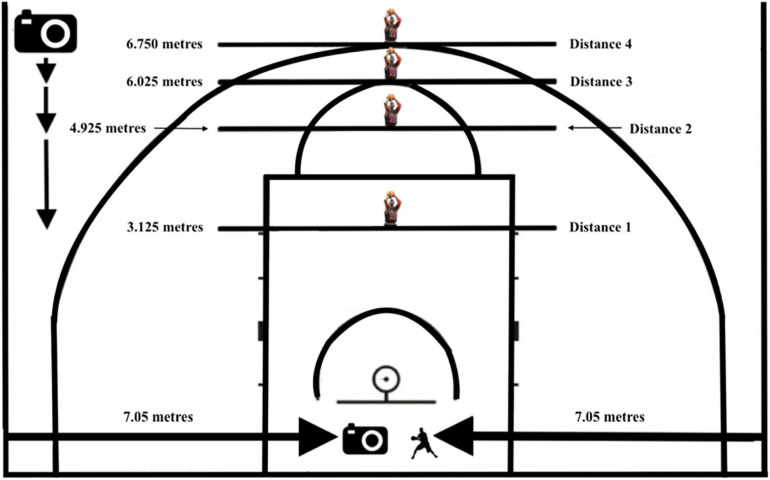
Schematic diagram of the experimental set-up showing the positions of the shooter (3.125, 4.925, 6.025, and 6.750 m), the side view camera (at each of the shooting positions), the front view (7.05 m from the sideline), and the passer (7.05 m from the sideline); front view camera and passer alternate depending on handedness of shooter.

The study was carried out in a standard basketball gym with the appropriate International Basketball Federation (FIBA) court markings, utilizing four shooting spots: the last hash mark before the free throw line (3.125 m), the length of an imaginary hash mark beyond the free throw line (4.925 m), the top of the free throw circle (6.025 m), and the three-point line (6.750 m) (see Figure 2). Prior to testing, the players were scheduled to shoot with another player, acting as an elite-level passer. Prior to the shooting session, all athletes, or parents for shooters under 18, were required to sign a consent to participate.

Each group of two players, upon entering the gym, went through basic demographic data collection, including age, position, last team played for, and height. Height was collected using a tape measure while the athlete wore shoes. Once this collection was done, the athletes were instructed to shoot ten game-like shots from each of the four spots, being videotaped the entire time. Each player determined the order of the four locations to shoot from first, with their partner making the passes. The warm-up resulted in a total of forty shots. No explanation was provided beyond this shooting task.

The warm-up shots were used to create a baseline which was later used to assess whether the experimental shooting depicted accurate results. The court camera set-up consisted of two cameras, a front view and a side view, to accurately assess which shooting motion the athlete used at each spot, “dipping” or “non-dipping.” The front view camera and the passer were aligned on either side of the basket (see Figure 2). The front view camera and passer were placed 7.05 m from each sideline, marked by a piece of tape. The camera was placed opposite to the passer, dependent on the handedness of the shooter. For example, if the shooter was left-handed, the passer was on the right side of the backboard, with the camera on the left side. The sideline camera enabled a view of 90° from the baseline to give an encompassed perspective of each player’s shooting motion.

**FIGURE 3:**
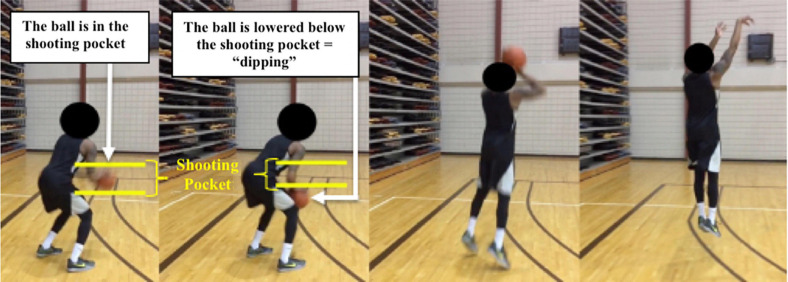
University player shooting form illustrating the “Dip.” First frame: the player caught the ball and has placed it into his shooting pocket, indicated by the yellow bars, Second frame: the ball has been lowered below the shooting pocket (yellow bars), meaning the player has “dipped” the ball. Frames 3, 4: the player finishes the entire shooting motion.

After the warm-up was completed, demonstrations of the expected movements for the “dip” and “non-dip” shooting motions were given by the principal investigator. Three key points of the “dip” were emphasized by the researcher (see Figure 3):

1. The ball must enter the shooting pocket after being caught from a pass.
2. The ball must be lowered below this shooting pocket, resulting in the shooting fingers and arm changing direction, indicated by at least a 90° increase between the elbow and wrist angles.
3. Keeping the ball in the shooting pocket while lowering the legs, without any change of direction from the fingers or arm, would not be considered a “dipped” shot.

After the athletes confirmed they were comfortable with the shooting motions, they were asked to select from a list of 18 sets of randomized numbers representing the four shooting locations and a list of 18 sets of randomized sequences of “dips” and “non-dips.” These lists were generated by the principal investigator using a “Research Randomizer” program online (Urbaniak and Plous, 2017). Once the order of location was established, the first player would start at their first distance. A combination of 20 “dipped” and “non-dipped” shots were to be taken at each distance. Before each shot, using the pre-selected randomized pattern, the shooter was informed which shooting motion, “dipped” or “non-dipped,” was to be executed. The player who was passing the ball to the shooter was instructed to pass in a manner similar to game conditions. Once twenty shots were taken, a 30 s break occurred. During this time, the research assistant, a current high school head coach, moved the camera to the next spot and watched the sequence to confirm all the shots were done correctly. Once confirmed, the next 20 shots could be taken. Once the first player’s 80 shots were taken, 20 shots at four spots, then the players switched positions so that the shooter became the passer and the passer became the shooter. The second player would then go through the same process using their pre-selected order. The shots were all recorded by the principal investigator. However, the shot was not tallied as “made” or “missed” but rather on a shot quality scale from 1 to 6, similar to the scale used by Hardy and Parfitt (1991), ranging from a 6 being a “swish” and 1 being an “air ball” (see Table 1). From a coach’s perspective, on the shot quality scale, made baskets were shown by a score between 4 and 6, while a missed basket was shown by a score between 1 and 3. The entire sequence resulted in a player shooting 40 warm-up shots and 80 recorded shots. Total testing time was approximately 20–25 min per player.

**TABLE 1:** Six-point shot quality scale with descriptions of visual representation.

**Scale value**	**Description**	**Made/missed**
6	“Clean” basket (“swish”)	MADE
5	Rim and in	MADE
4	Backboard and in (“bank shot”)	MADE
3	Rim and out	MISSED
2	Backboard and out	MISSED
1	Complete miss (“air ball”)	MISSED

*Hardy-Parfitt Scale starts at “0,” this scale starts at “1.”*

### Statistical Analysis

The shooter accuracy score was the cumulative shot quality value of each individual shot. A player could achieve a maximum average of six points per shot or a minimum of one point per shot, with the average used for the final score and statistical analysis. The athletes took ten warm-up shots at each spot which were used as a baseline to assess the quality of the experimental shots. The experimental shots that mirrored the player’s natural shooting motion, such as a “dipper” and his “dipped” shots, were compared to assess whether or not the shots fit the player’s pattern of accuracy using a paired-samples *t*-test. Using this type of test helped compare two means from the same group, seeing whether or not a difference existed. If no statistical difference existed between the warm-up and experimental shots, then the group’s results could be seen as more reliable. A 2 × 3 mixed model repeated measures analysis of variance (RMANOVA) was used to analyse the data of this study. There were three independent variables: whether or not the shot was “dipped” (shot type); the shooter’s natural shooting motion (shooter type); and shot distance. There was one dependent variable: the 6-point shot quality scale. The two repeated measures were: whether or not the shot was “dipped,” and the shot distance.

The data set was analyzed using the Statistical Package for the Social Sciences (SPSS), International Business Machines Corporation, Armonk, NY, United States. Any identified interactions were looked at using a paired-samples *t*-test to parse out the statistical significance.

### Bias

There were several points in the study where biases could impact the result. In the initial set-up, high school athletes were asked to shoot from the university three-point line (6.750 m), rather than the high school three-point line (6.250 m). The change in position was not seen as an issue simply because all of these elite high school athletes were training to make a university team in the coming years, and in most cases, had already trained at that distance for several years. Additionally, any effects from the unfamiliarity with the gym space were mitigated by testing players in their home gym. The location of the front view camera being on the opposite side of the basket from the passer helped reduce lateral bias.

The principal investigator was a university basketball coach who encouraged the “dip” in some of his players to help their accuracy. The potential confirmation bias was addressed by the randomness of the order of shot type, and order of the distances. In addition, another coach, the research assistant, confirmed that a player “dipped” or “non-dipped.”

### Sample Size

In order to calculate the sample size needed, the accuracy of shots for each group of players within their competitions was calculated. Using field goal percentage, the ratio of made shots compared to total shots, a better understanding of team accuracy was determined. Throughout the 2016/2017 season, the University of Manitoba Bisons maintained a 45.00% field goal percentage, making 796 successful shots while attempting 1,769 shots. During the 2017 Canada Summer Games, the Manitoba provincial team scored 154 shots and attempted 358 shots, resulting in a field percentage of 43.01%. During the 2016 Manitoba Games, the Winnipeg Gold regional team scored 126 shots and attempted 314 shots, resulting in a field percentage of 40.13%. In order to get an average high school percentage, the two sets of shots made and attempted were combined. The result was a high school field goal percentage of 41.67%. In order to not overestimate, a success rate of 42% was used, lower than the calculated 44.08% of all the teams. A proposed 19% while using the “dip” was used, based on the results of a pilot study prior to the present study. Assuming an α of 0.05, since the main hypothesis was one-tailed, and 80% power to detect a statistically significant difference between “dipped” and “non-dipped” shots was used. The sample size required was 24 athletes in total. Additionally, a literature review of jump shot studies demonstrated that the sample size was satisfactory (Miller and Bartlett, 1993, 1996; Keetch et al., 2005; Okazaki and Rodacki, 2012).

### Participant Data

Over the scope of the study, each player entered and completed the study fully. The overall sample population was 36 athletes. The players were classified as “dippers” or “non-dippers” (university “dippers,” *n* = 10; university “non-dippers,” *n* = 8; high school “dippers,” *n* = 13; and high school “non-dippers,” *n* = 5). The demographics were collected (see Table 2) and the mean accuracy scores tabulated (see Table 3). This sample was deemed a sample of convenience by the principal investigator because of his involvement in the coaching community.

**TABLE 2:** Demographics of the high school and university basketball players.

		**Dipped**			**Non-dipped**		
	**Factor**		**Average**	**Standard deviation**		**Average**	**Standard deviation**
High school players	*n*	13			5		
	Age (year)		16.6	1.0		17.0	0.7
	Year of Experience		6.4	2.3		6.0	1.0
	Height		187.2 cm	11.1 cm		185.0 cm	7.3 cm
			6′1.7″	44″		6′0.9″	29
University players	*n*	10			8		
	Age (year)		20.1	1.0		20.5	2.3
	Year of experience		10.8	2.6		11.5	1.8
	Height		198.3 cm	8.9 cm		194.4 cm	3.9 cm
			6′2.1	35″		6′4.6″	1.6″

**TABLE 3:** Summary of means in shooting data.

	**Means**		
	**Distance and shot type**	**Mean**	**Standard deviation**
High school “dippers” *n* = 13	Distance 1 and “dipped”	4.9	0.46
	Distance 2 and “dipped”	4.4	0.49
	Distance 3 and “dipped”	4.3	0.41
	Distance 4 and “dipped”	4.4	0.59
	Distance 1 and “non-dipped”	4.6	0.62
	Distance 2 and “non-dipped”	4.1	0.44
	Distance 3 and “non-dipped”	3.8	0.57
	Distance 4 and “non-dipped”	3.5	0.51
High school “non-dippers” *n* = 5	Distance 1 and “dipped”	4.6	0.68
	Distance 2 and “dipped”	4.3	0.69
	Distance 3 and “dipped”	4.0	0.30
	Distance 4 and “dipped”	4.0	0.60
	Distance 1 and “non-dipped”	4.4	0.74
	Distance 2 and “non-dipped”	4.1	1.01
	Distance 3 and “non-dipped”	3.7	0.50
	Distance 4 and “non-dipped”	3.4	0.50
University “dippers” *n* = 10	Distance 1 and “dipped”	5.0	0.48
	Distance 2 and “dipped”	5.0	0.44
	Distance 3 and “dipped”	4.5	026
	Distance 4 and “dipped”	4.4	0.39
	Distance 1 and “non-dipped”	4.3	0.41
	Distance 2 and “non-dipped”	4.4	0.42
	Distance 3 and “non-dipped”	4.0	0.57
	Distance 4 and “non-dipped”	4.0	0.47
University “non-dippers” *n* = 8	Distance 1 and “dipped”	5.1	0.42
	Distance 2 and “dipped”	4.7	0.49
	Distance 3 and “dipped”	4.4	0.38
	Distance 4 and “dipped”	4.3	0.57
	Distance 1 and “non-dipped”	4.8	0.55
	Distance 2 and “non-dipped”	4.5	0.61
	Distance 3 and “non-dipped”	4.3	0.39
	Distance 4 and “non-dipped”	4.1	0.59

### Main Results

**FIGURE 4:**
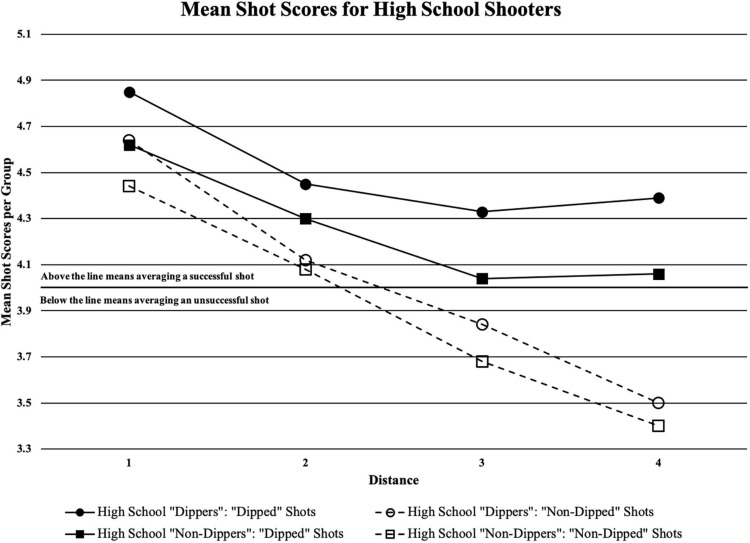
Mean shot quality scores from each of the two shooter types (“dipped” or “non-dipped”) high school players at each of the four distances; black horizontal line is the cut-off point for when a certain group would be averaging a successful shot (if above the line) or averaging an unsuccessful shot (if below the line).

**FIGURE 5:**
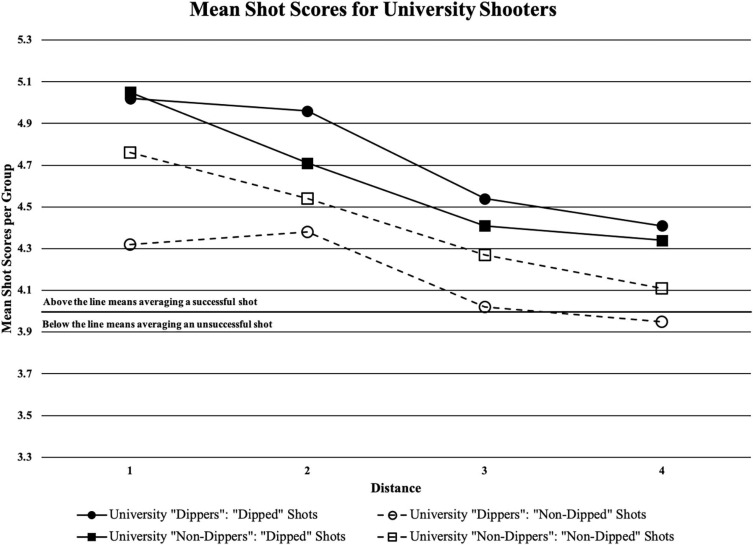
Mean shot quality scores from each of the two shooter types (“dipped” or “non-dipped”) university players at each of the four distances; black horizontal line is the cut-off point for when a certain group would be averaging a successful shot (if above the line) or averaging an unsuccessful shot (if below the line).

Table 4 shows the main effects of the study with shot type (“dipped” vs. “non-dipped”) being statistically significant as well as distance (the four shooting locations). There were statistically significant main effects of shot type in high school shooters (*F*_1_,_17_ = 27.608, *p* = 0.000), and in university shooters (*F*_1_,_17_ = 53.081, *p* = 0.000). From a coaching perspective, the mean accuracy score of high school shooters using the “dipped” shooting motion and the “non-dipped” shooting motion, respectively was 4.79 ± 1.24 and 4.57 ± 1.29 for distance 1; 4.41 ± 1.41 and 4.11 ± 1.36 for distance 2; 4.25 ± 1.39 and 3.80 ± 1.33 for distance 3; and 4.29 ± 1.43 and 3.47 ± 1.29 for distance 4 (see Figure 4). The mean accuracy score of university shooters using the “dipped” shooting motion and the “non-dipped” shooting motion, respectively was 5.03 ± 1.16 and 4.52 ± 1.28 for distance 1; 4.85 ± 1.24 and 4.45 ± 1.29 for distance 2; 4.48 ± 1.34 and 4.13 ± 1.29 for distance 3; and 4.38 ± 1.35 and 4.02 ± 1.29 for distance 4 (see Figure 5).

**TABLE 4:** Main effects and interactions of the present study.

	**Within-subjects effects**	**df**	***F*-statistic**	**Significance**
High school shooter				
	Shot type	1	27.608	0.000*
	Shot type × shooter type	1	0.735	0.404
	Distance	3	14.087	0.000*
	Distance × shooter type	3	0.112	0.953
	Shot type × distance	3	4.080	0.012*
	Shot type × distance × shooter type	3	0.113	0.952
University shooters	Shot type	1	53.081	0.000*
	Shot type × shooter type	1	10.995	0.004*
	Distance	3	16.102	0.000*
	Distance × shooter type	3	0.714	0.549
	Shot type × distance	3	0.448	0.720
	Shot type × distance × shooter type	3	0.155	0.926

*Significance at an α of 0.05.

There were main effects of distance in high school shooters (*F*_3_,_17_ = 14.087, *p* = 0.000) and university shooters (*F*_3_,_17_ = 16.102, *p* = 0.000). These results implied that shooting accuracy was impacted by distance. Specifically, for high school shooters, shot quality at distance 1 was better than distance 2, 3, and 4 (*p* = 0.005, 0.000, and 0.000, respectively), while shot quality at distance 2 was better than distance 3 and 4 (*p* = 0.047 and 0.001, respectively) using a paired-samples *t*-test. Conversely, for university shooters, shot quality at distance 1 was better than distance 3, and 4 (*p* = 0.000 and 0.000, respectively), while shot quality at distance 2 was better than distance 3 and 4 (*p* = 0.047 and 0.001, respectively) using the same test.

### Interactions

**FIGURE 6:**
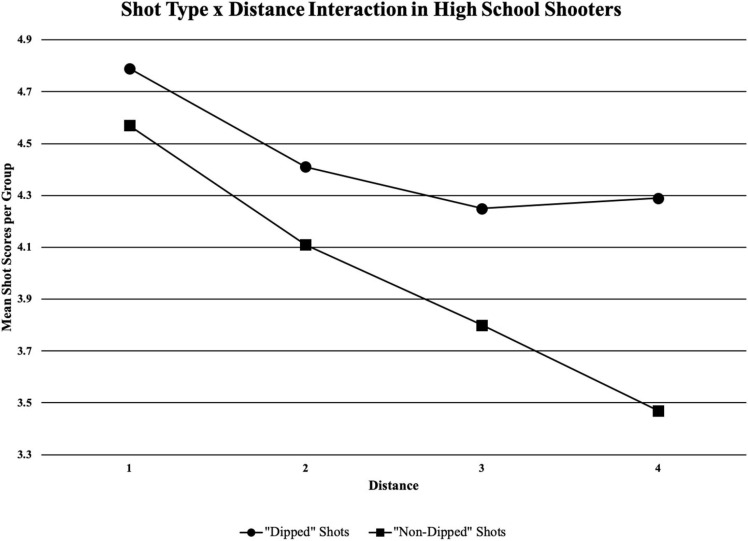
Shot type (“dipped” and “non-dipped”) and distance interaction in high school shooters; significant results for “dipped” shots between distance 1 and 2 (*p* = 0.009); distance 1 and 3 (*p* = 0.002); distance 1 and 4 (*p* = 0.004); significant results for “non-dipped” shots between distance 1 and 2 (*p* = 0.002); distance 1 and 3 (*p* = 0.000); distance 1 and 4 (*p* = 0.000); distance 2 and 3 (*p* = 0.011); distance 2 and 4 (*p* = 0.000); distance 3 and 4 (*p* = 0.008).

Within the analysis, there were interactions with both the high school and university shooters (see Table 4). For the high school shooters, there was an interaction between shot type and distance, meaning that “dippers” and “non-dippers” were combined to analyse the effect distance had on shot quality using a paired-samples *t*-test. There were statistically significant results for “dipped” shots occurring between distance 1 and 2 (*p* = 0.009); distance 1 and 3 (*p* = 0.002); and distance 1 and 4 (*p* = 0.004). There was no statistically significant difference between distance 2 and 3 (*p* = 0.141); distance 2 and 4 (*p* = 0.212); and distance 3 and 4 (*p* = 0.365). There were statistically significant results for “non-dipped” shots between distance 1 and 2 (*p* = 0.002); distance 1 and 3 (*p* = 0.000); distance 1 and 4 (*p* = 0.000); distance 2 and 3 (*p* = 0.011); distance 2 and 4 (*p* = 0.000); and distance 3 and 4 (*p* = 0.008). From a coach’s perspective, as distance increased, accuracy decreased, with the exception of “dipped” shot’s accuracy leveling off after distance 2 (see Figure 6).

**FIGURE 7:**
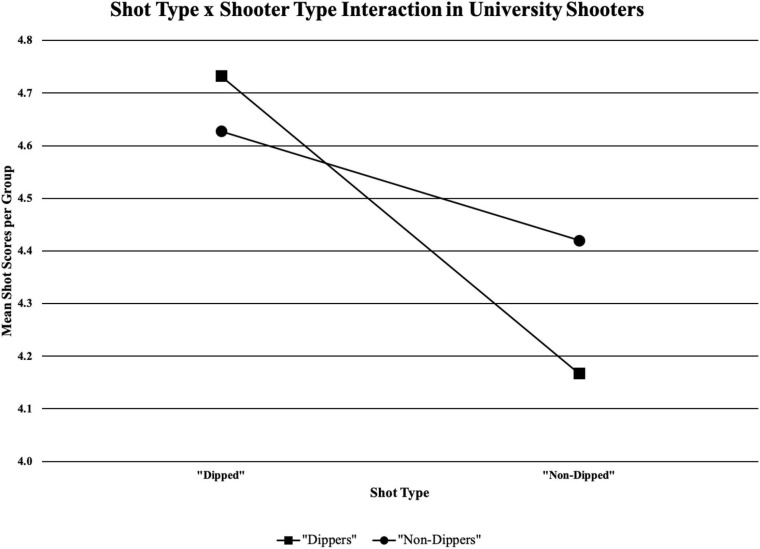
Shooter type (“dipper” and “non-dipper”) and shot type (“dipped” or “non-dipped”) interaction in university shooters; significant results for “dippers” using the “dip” compared to the “non-dip” shooting motion (*p* = 0.000); significant results for “non-dippers” using the “dip” compared to the “non-dip” shooting motion (*p* = 0.023).

For the university shooters, there was an interaction between shot type and shooter type (see Table 4). Distances were combined to analyse the effect natural shooting motion had on shot quality using a paired-samples *t*-test. There were statistically significant results for “dippers” using the “dip” compared to the “non-dip” shooting motion (*p* = 0.000). There were also statistically significant results for “non-dippers” using the “dip” compared to the “non-dip” shooting motion (*p* = 0.023) (see Figure 7).

## Discussion

The present study implies that the “dip” increases the accuracy of both the “dipper” and “non-dipper” at the high school and university levels. The scale discerns both whether or not the shot is made, but also identifies the quality of the shot. This scale also shows that the higher the group averages, the better the shot quality. The four groups, university “dippers,” university “non-dippers,” high school “dippers,” and high school “non-dippers,” all average a successful shot, represented by averages above four, when using the “dipped” shooting motion. Conversely, three of the four groups, university “dippers,” high school “dippers,” and high school “non-dippers,” average an unsuccessful shot, represented by averages below four, when using the “non-dipped” shooting motion. These results mean that only the university “non-dippers” average a successful shot using the “non-dipped” shooting motion.

In basketball, where accuracy is seen as an important factor for winning, the implication is that “dipping” the ball after catching would be an advantageous decision for all players to use in their jump shot shooting motion. The player’s natural shooting motion does not have an impact on whether or not the “dip” is effective as all four groups experience increased accuracy. The university “non-dippers” were the only group who had less of a benefit from “dipping,” but even they have increased accuracy and shot quality. This study implies that teaching the “dip” may be an effective way to increase accuracy is appropriate for elite level basketball players.

### Analytics of Basketball

In basketball, similar to other sports, the current approach to the game is becoming dependent on data analytics in order to improve every facet of the game. Situationally, the catch and shoot shot is seen as more accurate when compared to the dribble pull-up (Shea, 2014). During the 2013/2014 NBA season, the effective field goal percentage of catch and shoot shots was 52% while the effective field goal percentage of dribble pull-ups was 41% (Shea, 2014). Using the “dip” helps to mitigate some of the effects of distance on accuracy, particularly for high school shooters, implying the movement’s importance. Using the “dip,” and increasing accuracy, will also be more important as the game of basketball moves to a “three and key” style of play. This style emphasizes three-point shots, which were affected by the “dip” positively, and those in the key, mainly dunks and lay-ups.

Goldsberry (2012) evaluates the spatial accuracy of individual players all over the court. He suggests the use of the metric “range percentage” to better capture shooting ability (Goldsberry, 2012). The metric is calculated by dividing the basketball court into 1,284 scoring regions and highlighting which of those regions the athlete averages more than one point per attempt. For example, in the 2010/2011 NBA season, Steve Nash averages more than one point per attempt in 406 regions, meaning that his range percentage was 31.6%, the highest of the season. He is followed by Ray Allen. They are the only two players above 30%, suggesting that theirs was the best shooting ability during that season. While no metric may be perfect, this approach helps demonstrate who is a high quality shooter. Marty and Lucey (2017), and Marty (2018) further discuss variables, such as left-right value, and depth consistency, tailoring them to demonstrate the shot quality. The more accurate the shooter, the better these values appear. The majority of basketball analytics end with the offensive contribution of a player. Therefore, the accuracy of a player, as demonstrated by Goldsberry (2012), Marty and Lucey (2017), and Marty (2018) are paramount for his overall positive evaluation. “Dipping” the basketball yields the most desirable results according to the present study.

### Theoretical Considerations

The warm-up shots are used in a way that mimics the study and helps create a baseline for the players. This method allows for confirmation that the player did or did not have a bad shooting effort. If the player has a bad shooting effort, then the data is harder to trust. Inconsistent warm-up methods are a way that may affect overall study results. Methods such as participants conducting their own 10-min warm-up (Okazaki and Rodacki, 2012; Podmenik et al., 2017), having no warm-up (Myrtaj, 2012), or having the participant create their own warm-up (Liu and Burton, 1999) are all ways to negatively impact athlete readiness for the experimental shots. Therefore, the uniqueness of the warm-up is viewed as being more supportive of the current results.

The quality of the shot is the chosen evaluation, rather than purely whether or not the ball went in the basket. A player learning a new shooting motion might experience lower shooting percentages but a strong shooting form, implying that players who naturally did not “dip” have the potential to learn to “dip” without much effect on their shooting percentage initially, increasing their ability as they train the motion more often. Therefore, the training of the “dip” earlier in an athlete’s career can provide more of an advantage in using the “dipped” shooting motion. The high school shooters experienced a much larger difference between “non-dipped” and “dipped” shots, especially from farther distances. This difference suggests that there may be a “scaling effect,” meaning that small improvements to an athlete’s biomechanics and movement patterns may yield smaller benefits for the more “elite” player.

According to Nordland (2018), when a player “dips” the ball, there are three levels where the ball ends up: chest, waist, and thighs. This ending position of the backswing of the jump shot is seen as the “dip” position. From the “dip” position, according to Rick Penny, a shooting coach, there are three distinct shooting motions: “head pause,” “catapult,” and “one-motion” (Penny, 2016). Each shooting technique is using the “dip” because of the increase in wrist and elbow angles being greater than 90°. A player that uses the “head pause” shooting motion has a “dip” position near his waist. This motion results in a change in elbow angle of approximately 10°–15°, while the wrist angle would be between 75° and 90°. A player that uses the “catapult” shooting motion has a “dip” position near his thighs. This motion results in a change in elbow angle of approximately 45°, while the wrist angle would be greater than 45°. A player that uses the “one-motion” shooting motion has a “dip” position near his chest. This motion results in no or little change in the elbow angle, while the wrist angle would be 90°. These specifications imply are that there are many successful “dipping” motions, but a player must identify the motion that fits best with their shooting rhythmicity. Once this rhythm is achieved, then the quality of training and development increases, resulting in the athlete’s comfort and improvement in accuracy.

In order to incorporate the “dip” into a player’s jump shot, there are several practice methods that can be utilized. Firstly, developing an analogy for the “dip” will aid in the learning process by making the movement implicit (Masters, 2000). Secondly, athletes can utilize mental training techniques, such as mental rehearsal and self-talk, to improve comfort levels with the “dipping” shooting motion. Lastly, two types of practice should be used to improve the “dip.” Blocked practice, the repetition of the skill until some improvement is seen at a specific distance, should be used until the skill feels natural (Ragan, 2014, October). Random practice, practicing multiple skills in a random order with limited repetitions of the same skill, should be used to better retain the skill for long-term use (Ragan, 2014, October). Using these techniques in concert are important because many coaches suggest that block practice is best for those learning a new skill and random practice is best for those mastering a skill (Oliver, 2017).

### Strengths and Limitations

The present study has strengths and limitations. Within the scientific community, the “dip” has not been investigated and only anecdotal evidence provides support for the motion. The strengths of this study relate to the ability to experimentally demonstrate the “dip” and its effectiveness. Firstly, the isolation of the “dip” as the main contributor to a player’s accuracy is important because it eliminates concerns over distance or “dippers” naturally being better shooters. Secondly, the result provides coaches with scientific evidence that the “dip” increases accuracy and provides recommendations for incorporating into the player’s shooting forms. Lastly, the study is a template for future studies to address that the speed of the shot needs to be effective against defenders. In terms of limitations the sample should have equal numbers of athletes being “dippers” to “non-dippers.” This inequality is most prevalent in high school shooters, with only five “non-dippers.” Additionally, in a perfect situation, all playing styles are present, from the true center to the playmaking guard. This representation helps identify which players will truly benefit most from the “dip.” Lastly, one major limitation, however, is that even though sample size was calculated, the study was conducted with a convenience sample. This may have introduced selection bias to our results as players who shoot better and are more confident, may have volunteered.

## Conclusion

The results of the present study indicate that the “dip” increases the accuracy of all shooter types from every distance for both high school and university shooters. Anecdotally, players who use the “dip” often speak about how the motion improves their rhythm while those who do not use the “dip” suggest it slows their release time. These are important to consider in future coaching of the jump shot.

The present study creates a foundation for future studies on the effectiveness of the “dip.” From a coaching perspective, one might suggest that “dipping” could lead to the ball being stolen or blocked. Additional research needs to be conducted on release times and other parameters specifically to provide a basis on whether to use the “dip” beyond accuracy and address this concern. The laboratory setting is how the present study was conducted; therefore, it would be advantageous to observe games to determine when elite shooters use the “dip” and if accuracy improves in a game setting. For younger athletes, additional research needs to be conducted to see if there is more value in using the “dip” earlier in a basketball player’s career. Lastly, for elite athletes, additional research needs to be conducted to see if the scaling effect is outweighed by the amount of time needed to train the “dip” at a certain age.

## Data Availability Statement

The raw data supporting the conclusions of this article will be made available by the authors, without undue reservation.

## Ethics Statement

The studies involving human participants were reviewed and approved by the Education/Nursing Research Ethics Board. Written informed consent to participate in this study was provided by the participants’ legal guardian/next of kin.

## Author Contributions

The author confirms being the sole contributor of this work and has approved it for publication.

## Conflict of Interest

The author declares that the research was conducted in the absence of any commercial or financial relationships that could be construed as a potential conflict of interest.
